# Genetic Evaluation and Use of Chromosome Microarray in Patients with Isolated Heart Defects: Benefits and Challenges of a New Model in Cardiovascular Care

**DOI:** 10.3389/fcvm.2016.00019

**Published:** 2016-06-14

**Authors:** Benjamin M. Helm, Samantha L. Freeze

**Affiliations:** ^1^Department of Medical and Molecular Genetics, Indiana University School of Medicine, IU Health, Indianapolis, IN, USA; ^2^Department of Pediatrics, Indiana University School of Medicine, IU Health, Indianapolis, IN, USA

**Keywords:** chromosome microarray, congenital heart defects, copy-number variation, genetic counseling, clinical genetics

## Abstract

Congenital heart defects (CHDs) are common birth defects and result in significant morbidity and global economic impact. Genetic factors play a role in most CHDs; however, identification of these factors has been historically slow due to technological limitations and incomplete understanding of the impact of human genomic variation on normal and abnormal cardiovascular development. The advent of chromosome microarray (CMA) brought tremendous gains in identifying chromosome abnormalities in a variety of human disorders and is now considered part of a standard evaluation for individuals with multiple congenital anomalies and/or neurodevelopmental disorders. Several studies investigating use of CMA found that this technology can identify pathogenic copy-number variations (CNVs) in up to 15–20% of patients with CHDs with other congenital anomalies. However, there have been fewer studies exploring the use of CMA for patients with isolated CHDs. Recent studies have shown that the diagnostic yield of CMA in individuals with seemingly isolated CHD is lower than in individuals with CHDs and additional anomalies. Nevertheless, positive CMA testing in this group supports chromosome variation as one mechanism underlying the development of isolated, non-syndromic CHD – either as a causative or risk-influencing genetic factor. CMA has also identified novel genomic variation in CHDs, shedding light on candidate genes and pathways involved in cardiac development and malformations. Additional studies are needed to further address this issue. Early genetic diagnosis can enhance the medical management of patients and potentially provide crucial information about recurrence. This information is critical for genetic counseling of patients and family members. In this review, we review CMA for the non-genetics cardiology provider, offer a summary of CNV in isolated CHDs, and advocate for the use of CMA as part of the cardiovascular genetics evaluation of patients with isolated CHDs. We also provide perspective regarding the benefits and challenges that lie ahead for this model in the clinical setting.

## Introduction

Congenital heart defects (CHDs) are a common group of human malformations with significant morbidity and economic impact (1–3). The prevalence of CHDs in the general population has increased with ongoing advancements in medical and surgical care so that survival to adulthood is relatively common (4). In fact, the population of adults with CHDs is now larger than the number of children with CHDs. Despite the birth incidence of CHDs remaining relatively stable over the last half-century, the true global prevalence of CHDs is likely underestimated (2, 5). As more individuals with CHDs survive and reach reproductive age, questions regarding heritability, etiology, and recurrence risks will be common.

The majority of all CHDs are isolated or non-syndromic, but about 20–30% of infants with CHDs have extracardiac malformations (6). These cases often constitute well-known chromosomal and single-gene syndromes (e.g., trisomy 21, trisomy 18, and Noonan syndrome). However, complex rare diseases with CHDs and multiple congenital anomalies may remain undiagnosed, despite expert evaluation and/or the use of genetic testing. The underlying causes for the vast majority of CHDs remain unknown, especially in the case of apparently isolated or non-syndromic CHDs.

Approximately 20–30% of CHDs can be attributed to a single identifiable genetic or environmental cause (6–8), while the remaining cases are thought to be multifactorial. Examples of environmental risk factors include maternal disease (like maternal hyperphenylalaninemia, rubella, diabetes) and fetal teratogens (like alcohol, retinoic acid, and lithium) (9–11). CHDs are genetically heterogeneous, with numerous confirmed or proposed genetic risk factors, including single-gene variation, aneuploidy, chromosome rearrangements, and chromosome deletions/duplications. There are at least 55 human genes implicated in CHDs, but over 500 have been identified in mouse models (12). It is likely that the same magnitude will be eventually identified in humans. However, it is estimated that about 70–80% of CHDs have an unknown or multifactorial basis (13, 14). The complexity of genetic contributions probably reflects the complexity of cardiac development, and it is accepted that CHD development is influenced by multiple genetic (and environmental) factors. A multifactorial etiology emphasizing genetic contributions has been proposed for CHDs based on recurrence risk data (~1–4% across all lesions) and that the fact that family history is a consistent risk factor for CHDs (15–21). These recurrence risks generally increase as the number of affected first-degree relatives increases. Heritability estimates have been relatively high for specific classes of CHDs, namely, the left ventricular outflow tract obstructions (LVOTO) (22–24). The available evidence suggests that most CHDs have some genetic basis, but this is complicated further by variable expressivity and incomplete penetrance, even in families with an identified gene mutation or chromosome abnormality predisposing to the development of CHDs. Additionally, variants in the same genes can result in a spectrum of cardiac phenotypes.

As more individuals with CHDs survive and reach reproductive age, questions regarding inheritance and recurrence risk become increasingly important for reproductive planning and counseling. The recent advent of genomic technologies like chromosome microarray (CMA) and next-generation sequencing are providing additional diagnostic ability and refining recurrence information. As knowledge of the genetic bases of CHDs increases, genetic evaluation, testing, and counseling will continue to be important parts of the management of patients with CHDs. Current understanding of the multifactorial basis of CHDs is growing but far from complete, and cytogenetic analysis remains a valuable tool in the evaluation of patients with CHDs.

## Cytogenetics and Chromosome Microarray for the Cardiologist: A Review

Chromosome analysis has been a standard for investigating causes for developmental delay/intellectual disability, autism spectrum disorder, and congenital anomalies (25, 26). However, standard chromosome analysis (i.e., karyotype) has an estimated 3% detection rate for pathogenic chromosome abnormalities. Conventional chromosome analysis detects well-known chromosome aneuploidies (like trisomies 13, 18, and 21 or Turner syndrome) in about 10% of cases of CHDs (27). The innovation of CMA technology has increased the detection of chromosome abnormalities thought to be causative in individuals with developmental delay and congenital anomalies from 3% to about 15–20% (25). Karyotype has a genomic resolution of ~5–10 million base-pairs (megabases, or Mb); chromosome anomalies smaller than this are not consistently or reliably detected. Current CMA platforms generally have a genomic resolution of ≥250 thousand base-pairs (kilobases, or kb), though some platforms may have a resolution down to individual genes (1 kb).

One evident challenge of this increased genomic resolution is that smaller chromosome variations that have unknown clinical significance can be identified (28). This contrasts conventional chromosome testing (karyotype) in which large imbalances that are detected are all likely pathogenic, and it is uncommon to identify variants of unknown significance. Due to the increased diagnostic ability of CMA, Miller et al. (25) suggested that they be used as a first-line test over standard karyotype – though there are certain scenarios in which karyotype may be an ideal test (balanced chromosome rearrangements, family histories with multiple miscarriages, and/or reduced fertility).

Chromosome microarray is ideal for detecting chromosomal imbalances and copy-number variations (CNVs) in patients with birth defects and early developmental impairments (25, 29, 30). CNVs are generally defined as chromosomal deletions or duplications that cannot be detected using traditional chromosome analysis, generally sized 1 kb or greater. These CNVs are also referred to as “microdeletions” and “microduplications.” Additionally, other chromosomal imbalances can be detected like gross aneuploidy and higher-order amplifications like triplications. Interpretation of the clinical significance of CNVs is typically based on the overall size, gene content, location of breakpoints, and deletion vs. duplication of a chromosome region. Because the clinical significance of many CNVs may be uncertain, the American College of Medical Genetics and Genomics published guidelines to assist in predicting the pathogenicity of CNVs (31). Importantly, when a dose-sensitive gene is involved in the CNV region, deletion or duplication may have profound effects on the function of the gene and its protein products and potentially affect other downstream gene functions.

Chromosome microarray is performed by two strategies: array-based comparative genomic hybridization (aCGH) platforms or by single-nucleotide polymorphism (SNP) platforms. Array-based CGH utilizes short DNA sequence oligonucleotide probes, whereas SNP-based arrays use SNPs as probes. SNP microarrays also provide genotype information by detecting allelic copies of single base-pairs throughout the genome. Loss/gain of oligonucleotide probes on the aCGH platform and loss/gain of SNPs on the SNP-based platform, both indicate deletions and duplications, respectively. Current CMA platforms may merge these two strategies in the form of “oligo-SNP” microarrays. It is imperative that ordering providers understand the benefits and limitations of CMA platforms and be able to interpret and communicate results to patients/families.

## Copy-Number Variation and CHD: A Review of the Literature

While many CNVs are associated with well-described genetic syndromes, the role of CNVs in the development of all CHDs is not entirely known at this time. A few examples of well-characterized syndromes with CHDs caused by CNVs include Williams syndrome (7q11.23 deletion), DiGeorge syndrome (22q11.2 deletion syndrome), and Smith–Magenis syndrome (17p11.2 deletion). It should be noted that these conditions typically include other congenital anomalies, dysmorphic features, and neurodevelopmental disorders. Assessment by a clinical geneticist should be standard in these and similar cases with CHDs due to the presence of congenital anomalies and/or developmental delay.

It is estimated that pathogenic CNVs are present in 15–20% of patients with CHDs and extracardiac features (32–35). The submicroscopic deletions and duplications associated with these syndromes generally are not detected by routine chromosome analysis; therefore, emphasizing the importance of CMA as a part of the diagnostic workup in patients with CHDs. Although CNVs play an important role in the development of genetic syndromes with CHDs, most CHDs do not occur in the context of a genetic syndrome. While the exact contribution of CNVs to isolated CHDs is unclear, studies show that ~4–14% of individuals with isolated CHDs have pathogenic or suspected pathogenic CNVs (36, 37), though others have suggested 3–10% (13, 38). Geng et al. (39) retrospectively reviewed 514 CHD cases that had CMA testing, contrasting the yields between isolated and syndromic cases. They found pathogenic or likely pathogenic results for 4.3–9.3% of isolated CHD cases. The yield was higher for syndromic cases when excluding aneuploidies. Additional large-scale studies are necessary to further specify and support these estimates.

The few studies that have investigated the contribution of CNV to the development of isolated CHDs are providing insight into additional genes and pathways involved in cardiovascular development and malformation. These studies can also provide additional understanding about heritability, recurrence, variable expression, and incomplete penetrance of CHDs in families. In the studies summarized in Table 1, there are examples of apparently isolated CHDs that were found to have CNVs overlapping known syndromic regions [e.g., 22q11.2 deletion and duplication, 16p11.2 duplication; see Silversides et al. (40)]. It is unclear if those patients had been evaluated for and/or diagnosed clinically with a genetic syndrome, or they had been unrecognized or only presented with mild features. These studies have not only provided additional information about candidate genes and pathways associated with CHDs or risk of CHDs but also highlight that even apparently isolated CHDs may actually be syndromic. This information can inform patient evaluation and may lead to early diagnosis, which can have positive impact on management and genetic counseling. Utility of genetic testing depends largely on accurate phenotyping of the CHD lesion and the presence of extra-cardiac features. Further studies with meticulous phenotyping and goals to assess broad classes of CHDs lesions should be undertaken to further refine this estimate. This also highlights the critical importance of involvement of clinical geneticists in the evaluation of seemingly isolated CHDs.

**Table 1:** **A summary of CNVs identified by CMA in non-syndromic CHDs reported in the literature**.

Study	CNVs (limited list)	Confirmed or putative candidate genes for CHDs noted by authors (limited list)	CHD types in study	Other notes
Thienpont et al. (41)	4q34 deletion		AS, TOF, CoA, VSD, truncus arteriosus, PS	
	5q35.1 deletion	*NKX2-5*		
	9q34.3 deletion	*NOTCH1*		
	22q11.2 duplication			
Erdogan et al. (36)	1q21.1 deletion	*GJA5*	Any CHD: majority were VSD; TOF, PS, CoA, ASD, AS, HLHS, and AVSD	The 17p11.2 deletion is causative for Smith–Magenis syndrome. Features of this disorder were not appreciated until after the test result
	2p22.3 duplication	*LTBP1*		
	17p11.2 deletion	*(See note in last column)*		
	22q11.2 duplication	*TBX1, CRKL*		
Greenway et al. (42)	1q21.2 deletion and duplication	*GJA5, PRKAB2, CHD1L, BCL9*	TOF	Study involved only subjects with TOF
	2p23.3 duplication	*ASXL2, KIF3C, RAB10*		
	3p25.1 duplication	*RAF1*		
	9q34.3 deletion	*NOTCH1*		
	20p12.2 deletion^a^	*JAG1*		
	22q11.2 deletion^a^	*TBX1, CRKL*		
Silversides et al. (40)	1q21.1 duplication	*GJA5*	TOF	Study involved only subjects with TOF
	1q32.2 deletion	*PLXNA2*		
	3p25.1 deletion	*RAF1*		
	7q21.11 deletion	*SEMA3E, SEMA3D*		
	7p15.3 deletion	*DNAH11*		
	7p22.2 deletion	*SNX8*		
	8p23.1 deletion^a^	*GATA4, ANGPT2*		
	8p23.3 duplication	*ARHGEF10*		
Soemedi et al. (37)	1q21.1 duplication	*GJA5*	TOF, ASD, VSD, CoA, complex left-sided defect, TAPVR	Other rare CNVs identified with unconfirmed candidate genes associated with cardiac development; TOF overly represented
	4q34 deletion	*HAND2*		
	5q14.1q14.3 duplication	*SSBP2, TMEM167A, VCAN, EDIL3*		
	5q35.3 duplication	*CNOT6*		
	8p23.1	*GATA4*		
Fakhro et al. (43)	1q32.3 duplication	*NEK2*	Heterotaxy with: D-TGA; dextrocardia; VSD, ASD, PAPVR; malposed great arteries, CoA	Study involved CHDs with heterotaxy
	2p25.1 duplication	*ROCK2*		
	3p24.1-p23 deletion	*TGFBR2, RBMS3, GADL1*		
	3p24.1 duplication	*TGFBR2, GADL1*		
	7q36.1 deletion	*GALNT11*		
	8p23.1 deletion	*GATA4*		
	9q34.11 duplication	*NUP188*		
Zhao et al. (44)	3q21.3 duplication	*PLXNA1*	ASD, VSD, PDA, TOF, Ebstein anomaly, tricuspid incompetence	Study involved 100 Han Chinese subjects
	16q23.1 duplication	*WWOX*		
	18q23 duplication	*NFATC1*		
	22q11.2 deletion^a^	*TBX1, CRKL*		

*AS, aortic stenosis; ASD, atrial septal defect; AVSD, atrioventricular septal defect; CHD, congenital heart defect; CoA, coarctation of the aorta; D-TGA, dextro-transposition of the great arteries; HLHS, hypoplastic left heart syndrome; PAPVR, partial anomalous pulmonary venous return; PDA, patent ductus arteriosus; PS, pulmonary stenosis; TAPVR, total anomalous pulmonary venous return; TOF, tetralogy of Fallot; VSD, ventricular septal defect*.

*^a^It was unclear if these reports included patients with clinical diagnoses of syndromic disorders (i.e., DiGeorge syndrome for 22q11/2 deletion or Alagille syndrome for the 20p12.2 deletion). It could be that these reports were either unrecognized syndromes or individuals who were mildly affected*.

## CMA for the CHD Population: Interpretation of Results

Chromosome microarray is recommended as a first-tier clinical genetic test in cases of isolated CHDs due to the relatively high rate of detection of pathogenic CNVs. Positive results in the patient with isolated CHDs can provide important information for practitioners and family members when making decisions regarding ongoing care and family planning. The negative CMA result can also be critical in guiding next steps for care and in limiting the differential for any given patient. Many well-described chromosomal conditions can be eliminated as diagnoses by a normal CMA result. This elimination can guide further genetic testing decisions and options for additional clinical testing to hone in on the exact diagnosis for the patient.

While implementing the use of CMA in the diagnostic evaluation of patients with CHDs has uncovered previously unknown pathogenic chromosome variation, it has also presented the unique challenges of interpreting variants of uncertain significance (VUS). Designation of VUS is typically reserved for deletions or duplications that have not been previously described, have not been seen in studied control populations, and for which there are incomplete data regarding genes in the affected region (45). Adding to this difficulty are the concepts of incomplete penetrance and variable expressivity. Some VUS results have been reported in the literature with highly variable phenotypic features due to variable expressivity, adding further complexity to the interpretation of the contribution of any given VUS to the phenotype of the patient. Incomplete penetrance of CNVs also complicates the recommendations and counseling provided to families, as accurate risk prediction for certain health concerns cannot be provided. Of particular concern is the patient with significant morbidity who inherits a CNV of unknown significance from a typical-appearing parent. This situation requires discernment from both the calling laboratory and the health care team in order to provide an accurate risk assessment to the “unaffected” parent as well as the affected child and the significance of the familial CNV. It may also be difficult to provide accurate recurrence risk information for reproductive decision-making if the contribution of the CNV to the affected patient’s phenotype is unclear. Parental studies should be offered in the event that a VUS is found in a child in order to aid in interpretation and significance of the CNV regardless of whether the parents have similar or dissimilar phenotypes. However, insurance coverage and justification of how this information will impact the parental medical management may prove to be difficult and require the adamant support from the health care team in order to secure insurance coverage. The likelihood of discovering a VUS should be outlined to the family as part of the pretest informed consent process.

Another challenge when using CMA in the CHD population is the “one-hit fallacy” or the notion that any specific CHD is caused by one particular genetic variation alone. CHD is a multifactorial disease caused by both environmental and genetic factors. The contribution of any one CNV to the overall risk for CHDs is difficult to assess. While ~20% of CHDs can be attributed to a known cause (syndromic, teratogenic, etc.), the vast majority of CHDs is non-syndromic, isolated defects exhibiting a multifactorial inheritance pattern. In any one case of isolated CHD, there may be multiple genes involved, each providing a minimal contribution to the patient’s risk, interacting with various environmental factors to form a complex model of CHD development.

One area in which health care providers can aid in the interpretation of a CNV is to provide accurate and thorough phenotyping prior to the completion of genetic testing. By performing CMA for a patient, a genome-wide net is cast in the hopes of finding an explanation for the patient’s particular phenotype. By casting such a wide net, results can often be complicated by overlapping clinical diagnoses and lack of genotype/phenotype correlations. CNVs must also be considered in the context of size, location, and gene involvement. Understanding of the clinical significance of a CNV involving genes that have yet to be well described or that have yet to be implicated in a particular phenotype can prove to be difficult. One example of distinguishing cardiac phenotypes is the presence of an atrial septal defect (ASD) and the classification of primum vs. secundum ASD. Primum ASDs are within the spectrum of atrioventricular canal defects, whereas secundum ASDs are a malformation of the atrial septum (46). This classification distinguishes the CHDs from a developmental perspective and can aid in the interpretation by narrowing focus on genes associated with the responsible developmental process. Accurate and specific phenotyping will require coordinated efforts from cardiologists and clinical geneticists.

The process of interpretation of CNVs is ongoing and constantly evolving. As CMA continues to be performed as a first-line test for patients with CHDs, CNVs classified as VUS will continue to present challenging clinical scenarios for health care providers. While it is important that both the laboratory and the health care team work together to interpret CNVs and assign appropriate labels of pathogenicity, it is also important to acknowledge current limitations in our understanding of the human genome and the contribution of variation to cardiovascular disease phenotypes. As our knowledge continues to increase, the opportunity to further refine and identify novel phenotypes presents an exciting challenge for the cardiovascular genetics community.

## The Importance of Genetics Care Provider Involvement with CHD

Our understanding of the association between CNVs and syndromic genetic diagnoses is increasing. There are many examples of newly described microdeletion and microduplication conditions with CHDs (47). Variable expressivity of these conditions and the generally small number of patients described in the literature can make it difficult to recognize associated features and make an accurate diagnosis. Even more well-described syndromes, such as DiGeorge syndrome, 1p36 deletion syndrome, and Williams syndrome, can go undetected for many years in patients with mild or variable presentations. Early involvement of the clinical genetics team provides the opportunity for earlier recognition of syndromic conditions, which can result in more comprehensive medical interventions and therapies as well as improved prognosis, compared to those patients who receive a syndromic diagnosis later in life. There is also increasing recognition that many delineated syndromes have broader phenotypic variability than previously thought (34, 35, 48). Many syndromes may not be recognized earlier in life due to absence of the “classic” defining features (49). CHD, which is present at birth, can provide a framework for the genetics provider to begin the process of creating a differential for the patient due to the higher prevalence of certain types of CHD lesions in certain genetic conditions (50, 51). CMA, as a first-line genetic test, can detect causative CNVs for many syndromic conditions with a CHD component well before other hallmark features of the diagnosis can be recognized. When CMA is negative, additional genetic testing, including sequencing of genes associated with known conditions and/or whole-exome sequencing, may be warranted for patients with multi-system involvement or features suggestive of a particular genetic condition.

Clinical geneticists and genetic counselors serve as valuable resources to family members of patients with CHD. Early syndromic recognition by the geneticist physician and continued follow-up by a genetic counselor can provide valuable information to the family regarding the anticipation of developmental delays and disabilities, available therapies, and social services that might benefit their child. Early diagnosis can also refine recurrence risk estimates and allow families to make informed reproductive planning decisions. Genetic evaluation and risk assessment can prove to be a powerful tool for empowering families to use genetic information to make informed health decisions. A unique role of the genetics team is the ability to clearly communicate familial risk for CHDs and recommendation of family screening protocols. First-degree family members with certain types of CHDs are at an increased risk to also have undetected CHDs. For example, LVOTO heart defects are understood to be a heritable class of defects, and family members have an increased risk of also having a CHD (22). Studies show that in up to 20% of cases, there is at least one other affected relative in the family with variability in type of LVOTO present. Therefore, screening by echocardiogram is recommended for all first-degree family members of someone affected with an LVOTO class of heart defect (23, 52).

An emphasis must be placed on the coordinated efforts of the cardiologist, clinical geneticist, and genetic counselor in the evaluation, management, and follow-up with patients with CHDs and their family members. This same approach should be used when considering CMA testing and interpretation in this population. A multidisciplinary approach provides a comprehensive care model for patients and families. Genetic testing through the use of CMA, even in patients with apparently isolated CHDs, can aid in delineation of diagnosis, accurate risk assessment for family members, and refinement of recurrence risk estimates for reproductive decision-making. Accurate phenotyping and diagnosis can improve patient outcomes and access to necessary evaluations, therapies, and social services. Genetic counseling and education can empower patients with CHDs and their relatives to use their understanding of the genetic basis of cardiovascular disease to in turn choose effective strategies for health maintenance and appropriate psychosocial coping mechanisms.

## Author Contributions

BH conceived this review article and provided 50% of the work presented in the manuscript. SF completed the remaining 50% of the manuscript. Both authors contributed significant effort in the writing process.

## Conflict of Interest Statement

The authors declare no conflicts of interest, financial or otherwise, in the writing of this manuscript. This is a focused review article that did not involve any human or animal subjects’ research and was exempt from IRB review.
